# Factors Influencing Sedentary Behaviour in Older Adults: An Ecological Approach

**DOI:** 10.3934/publichealth.2016.3.555

**Published:** 2016-08-15

**Authors:** Linna Tam-Seto, Patricia Weir, Shilpa Dogra

**Affiliations:** 1 School of Rehabilitation Therapy, Faculty of Health Sciences, Queen's University, Kingston, ON, Canada; 2 Faculty of Human Kinetics, University of Windsor, Windsor, ON, Canada; 3 Faculty of Health Sciences (Kinesiology), University of Ontario Institute of Technology, Oshawa, ON, Canada

**Keywords:** sitting, aging, physical inactivity, leisure time

## Abstract

Sedentary behaviour is negatively associated with several health outcomes and is particularly problematic among older adults. Knowledge translation tools and public health promotion strategies are needed; however, little evidence is available to inform framing of such tools or development of intervention programs. The aim of the present study was to use data on the perceptions of sedentary time and the programs or supports older adults identify as important for reducing their sedentary time, to inform knowledge translation strategies targeting this population. Focus groups were conducted with four groups of older adults (n = 26) at local seniors' centres (Ontario, Canada). Participants were 74 ± 8.5 years old and were engaging in both sedentary and physical activities in a social environment. Using the Ecological Model for sedentary time in adults, we categorized data into leisure time, household, transport and occupation domains. Intrinsic and extrinsic factors that worked to either discourage or promote sedentary behaviour were identified. Drawing on both groupings of data, results were synthesized to inform public health strategies on appropriate messaging and better uptake of programming and guidelines. For example, successful programs developed on the topic will need to include a social component and a mentally stimulating component, as these were identified as critical for enjoyment and motivation. It was clear from this analysis that sedentary time reduction strategies will need to consider the different domains in which older adults accumulate sedentary time.

## 1. Introduction

Sedentary time is associated with a variety of biopsychosocial outcomes in middle-aged (45–64 years) and older adults (65 years and older) [1]. Indeed, a growing body of evidence indicates that sedentary time is negatively associated with all-cause mortality [2], cardiometabolic disease [3], and functional ability [4] in older adults. It appears that prolonged sedentary time is particularly harmful, and that breaking up sedentary time is associated with better health outcomes [5]. Unfortunately, evidence suggests that 93.6% of older adults are sedentary for 8 or more hours per day [6]; this is consistent across many countries [7].

An ecological model proposed by Owen et al. [8] identified four domains in which adults are sedentary; these domains are household, leisure time, transportation, and occupation. Each of these domains is influenced by individual, social, organization/community, environmental, and policy levels. This model highlights the importance of understanding the physical and social contexts in which sedentary time is accumulated. A variety of different factors promote or discourage sedentary behaviour in each of these domains. By understanding these factors, strategies to reduce sedentary time in each of the four domains can be developed. Some domains may be particularly relevant to the study of sedentary behaviour in older adults. For example, occupation is likely to be a small or non-existent portion of the day among retired older adults, while leisure and household activities likely make up the bulk of the day. Understanding the patterns of sedentary time, and the domains in which sedentary time is accumulated, is important to inform public health messages for this population.

Evidence-based guidelines are readily available for physical activity promotion; however, physical activity levels remain suboptimal in older adults [9]. An important step when creating guidelines and public health messages around sedentary time is to understand the views of older adults. A recently published study using focus groups with older adults [10] showed that despite being sedentary, older adults do not identify with the term sedentary, and view it negatively. Thus, something as simple as referring to sedentary behaviour guidelines as sitting guidelines may significantly alter their uptake. As such, the aim of the present study was to use data on the perceptions of sedentary time and on the programs or supports older adults identify as important for reducing sedentary time, to inform knowledge translation strategies targeting this population.

## 2. Methods

### 2.1. Study Design and Method

This qualitative study is framed using phenomenology; a method that is used to understand participant experiences [11]. Four focus groups were conducted at separate locations on separate dates at the same time of year (December). The methodology for this study has been described in detail elsewhere [10]. All methods and communications were approved by the Research Ethics Board at the University of Ontario Institute of Technology.

The original purpose of the focus groups was simply to understand the perceptions of older adults regarding sedentary behaviour; these data have been published [10]. In addition, the investigators wanted to learn more to better understand how to reduce sedentary time among older adults. The probes used in each of the focus groups were: Describe what sedentary behaviour means to you; Describe the pros of the sedentary behaviours in which you participate; Describe the cons of the sedentary behaviours in which you participate; What types of programs do you feel may help reduce sedentary behaviour?; What type of support do you think would help increase weekly physical activity levels? Participants were provided with a description of sedentary behaviour that related to the accepted definition of activities completed in a seated or reclined position requiring low energy use [8],[12].

### 2.2. Participants

Community-dwelling older adults from four sites of a senior citizen's centre in a mid-sized city in southern Ontario, Canada, agreed to participate. Eligibility was limited to adults over the age of 55 years. There were no additional exclusion criteria. Staff at the seniors' centre identified groups engaging in sedentary activities and arranged for the research team to conduct focus groups with these individuals. The sample was 77% female aged 74 (± 8.5) years (n = 26). Participants self-reported engaging in 5.6 (± 1.0) hours of sedentary time per day based on the Physical Activity and Sedentary Behaviour Questionnaire [13] Approximately 60% of the sample was widowed and 47% had a high school education or less [10]. Participation in the study was voluntary and all participants provided written informed consent.

### 2.3. Research Team

The principal researcher and senior author of this paper has a PhD in Kinesiology and Health Sciences with a research specialty in the area of active ageing. She has also published on the subject of sedentary time in older adults. One of the co-authors acted as the moderator of the focus groups. She is a PhD student, an Occupational Therapist and a researcher in the area of mental health using qualitative methods. The other co-author on this paper is a researcher with expertise in successful aging and physical activity.

The assistant moderator for the focus groups was an undergraduate student conducting a research project under the supervision of the principle researcher; however she was not involved in this current paper. Neither of the moderators had previous experience with research in this content area.

After completion of each focus group, the research team debriefed on their personal experience of the focus group as well as any strategies that may inform subsequent sessions.

### 2.4. Data Analysis

Focus group transcripts were reviewed by each member of the research team. A consensus approach was taken in the directed content analysis of the data. Directed content analysis was guided by processes described by Hsieh and Shannon [14]. Initial coding categories were sedentary behaviour domains articulated in the Ecological Model by Owen and colleagues [8]. Analysis was completed by each of the authors. Codes were independently extracted by all members of the team in relation to identifying factors that either promote or discourage sedentary behaviour. This was done within the four domains of the ecological framework. Subsequently, the research team identified themes within each domain and across the factors that promote or discourage sedentary behaviour.

All themes and subthemes were organized into one of the four domains of sedentary behaviour: household, leisure time, transport and occupation [8]. Each of the three researchers defined the domains as per their previous research in related areas. These definitions were then discussed and a consensus was reached. While definitions for each of these domains exist, none to our knowledge are specific to older adults in a sedentary behaviour context. The household domain was defined as activities required to maintain one's home, such as indoor chores and yard work. The leisure time domain was defined as any activity that was done for personal enjoyment, i.e. in non-work time unrelated to household activities. The transport domain was defined as activities associated with public transportation, active transportation and driving. Finally, the occupation domain was defined as activities that were related to paid and unpaid work such as volunteering.

## 3. Results

Two main themes that fell into each of the four domains of the ecological model of sedentary behaviour emerged from the data [8]. These themes identified factors that either discouraged or promoted sedentary behaviour. Within each of these themes, subthemes emerged that further articulated the nature of these factors as being either intrinsic in nature or extrinsic in nature.

**Table 1.** Factors that Discourage and Promote Sedentary Behaviour in each of the Four Domains.

**Table:** (a) Factors that Discourage Participation

	Leisure Domain	Transportation Domain	Housework Domain	Occupation Domain
**Intrinsic**	Enjoyment	Motivation	Enjoyment	Enjoyment
	Companionship	Physical health		Motivation
	Mental stimulation			
	Motivation			
**Extrinsic**	Awareness of programs	Access		
	Physical health

**Table:** (b) Factors that Promote Participation

	Leisure Domain	Transportation Domain	Housework Domain	Occupation Domain
**Intrinsic**	Physical health			
	Aging attitudes			
	Financial costs			
	Lack of motivation			
	Enjoyment			
	Lack of companion-ship			
	Mental stimulation			
**Extrinsic**	Program access	Access		
	Awareness of programs	Transportation		
	Financial costs	Weather		
	Cultural/feeling accepted			

Promoters of sedentary behaviours were considered to be factors that support the participation or the decision to participate in sedentary behaviours. In contrast, factors that discourage sedentary behaviours were those that prevented or decreased the participation or decision to participate in sedentary behaviours. Subthemes were then derived, grouping codes as intrinsic and extrinsic factors for both.

### 3.1. Factors that Discourage Sedentary Behaviour

#### 3.1.1. Leisure Time Domain

A number of intrinsic factors that discouraged sedentary behaviour were identified when focus group participants discussed leisure time activities, including *enjoyment, companionship, mental stimulation* and *motivation*. Participants described that part of the joy of attending fitness classes was building friendships and meeting people:

“I met a lot of nice people. We even had dinner, um… we had like a little group going of-of ladies where you know, once a month we held a dinner at a, pot luck dinner at someone else's house.”

For *mental stimulation* participants described how being around others and participating in programs were mentally stimulating.

“It gives you uh… company. You got no four walls when you're here. You've got people. You're interacting. You're… stimulating yourself mentally.”

“And there's stimulation with all these exercise programs that they put on, also mental…”

For *motivation*, some participants described how individuals need to have an internal desire to engage in certain activities, particularly those that are located in the community as a great deal of effort is required to participate.

“I mean generally speaking when you're talking about seniors, I think even just, you know, they don't have to be physically like running and doing all of that stuff. But … when you have a senior come in to a center like this, … They're getting outta bed, they have to get dressed, they have to either you know, um… Walk or take a bus or get a taxi or get a ride or something. They gotta walk through the parking lot and walk in, you know…. We've had people huffing and puffing by the time they get in here with their walkers, but I mean, that's, that's already more exercise than they would have had sitting at home.”

Extrinsic factors identified included *awareness of programs* and *physical health*. Discussions around *awareness of programs* ranged from hearing about programs from friends and reading about them in local papers.

“It was the [local] paper that got me started. I had retired, and I says ‘what am I gonna do?’ And I saw in the paper … That's what started and I've been here, living in Senior Citizens since. That's my, almost my first home.”

Depending on the individual's *physical health*, their involvement in sedentary behaviours varied. Some participants indicated that existing health conditions required that they participate in physical, rather than sedentary activities:

“I've had many people come and say ‘Well my doctor said I need to … I need to do the swim ….swim program’ or whatever we offer … you know, people will come. I think it's, something that will push people…”

#### 3.1.2. Transportation Domain

*Motivation* and *physical health* were identified as intrinsic factors that discouraged sedentary behaviour. *Motivation* was apparent when older adults spoke about ways in which they added active transportation to their day: “And you purposely park your van, as far as you can”.

*Physical health* impacted the transportation domain and referred to ambulation and how that influenced engagement in sedentary behaviours:

“Yeah, bionic knees…But yeah, walking is wonderful, that's my biggest thing that I really like to do.”

The only extrinsic factor discouraging sedentary behaviour identified in the transportation domain was *access.* For example, those with access to a car had additional work to do.

“Wintertime I have a snow blower but there, there times when I have to shovel as well. So it's shoveling and snow blowing. My driveway's long so I can get three cars, length wise.”

#### 3.1.3. Household Domain

Based on the data, gardening was the main household activity described during the focus group discussion. *Enjoyment* was mentioned in the conversations about gardening and was the sole intrinsic factor discouraging sedentary behaviour:

“I love my gardening so that takes care of the spring time when you have to dig up and, and, and, and clean up your garden and plant new plants and stuff and then cutting the grass every week and I have cottage, so I have to do it there too.”

#### 3.1.4. Occupation Domain

The majority of the focus group participants were retired, thus their main occupation was volunteer work. When describing volunteer activities, factors that discouraged sedentary time were identified as *enjoyment* and *motivation*. Volunteer work was associated with being on the go and not having the time to sit down. *Enjoyment* was evident in the pride that volunteers had:

“I think that the people that are volunteers at, OSCC are a different, different group of people all together than you're gonna find in a retirement home or something like that because we're busy volunteering, we're doing things we're a different breed of people than anything else.”

*Motivation* was evident by the number of hours that volunteers were contributing to a variety of committees.

“You know, I put on my paper, in less than in about four and a half years, I had over twenty-seven hundred hours of volunteer time and then I volunteer three days a week, some days its four hours here.”

### 3.2. Promoters of Sedentary Behaviour

#### 3.2.1. Leisure Domain

There were a number of intrinsic promoters identified in the area of leisure including: physical health, aging attitudes, financial costs, lack of motivation, enjoyment, lack of companionship and mental stimulation. Physical health was described in all of the focus groups as a reason that participants decide to engage in sedentary behaviours.

“I have the problems that I have a lot of back issues and basically [sedentary activities are] the only type of activities that I can do. The joints give out! You can't. You just can't do it. Yeah. You can only push yourself so far!”

Associated with the physical changes that occur with aging were the individual and social *attitudes of aging*, which appeared to promote engaging in sedentary behaviours:

“I think, I think for one thing, the senior citizens part scares a lot of people away. I've often said, it should be just, plus fifty-five plus club or, you know Zoomers club. You know? Whatever it is because Senior Citizens, it just means old. It really does. Because you have to be a certain age.”

Although many of the programs offered at the centres are subsidized, participants indicate that *financial costs* to access the programs can also hinder people from joining which will further support the decision to engage in solitary sedentary behaviours. *Financial cost* is an intrinsic factor when the individual is required to prioritize how their limited income is going to be spent.

“And do you want me to tell you another thing? Money. It costs a lot to take part in almost everything here. The only thing that doesn't cost is coming here for canasta even euchre is only a couple of dollars, bingo a very little money. But any of those classes and things, a lot of people can't afford them.”

*Motivation, enjoyment, companionship* and *mental stimulation* were all previously identified as intrinsic factors discouraging sedentary behaviours. These same themes were also identified as intrinsic promoters of sedentary behaviours. One participant indicated that she had purchased a variety of simple exercise equipment to continue what she had learned in a fitness class but admitted that she did not have the motivation to do them on her own.

“I bought those weights and those stretchy bands, but they're not doing a darn thing. They're on the coffee table. They haven't done, done very much at all. We took stretch and sculpt you know, and…”

Related to *motivation* is the idea of enjoyment participants identified in their preferred activities, many of them being sedentary in nature such as using the computer or knitting, and conversely a lack of motivation to be physical. *Companionship* was also identified as a promoter of sedentary behaviour, more specifically the lack of companionship:

“Yeah well I live on my own. So yeah, a lot of the activities I do is sitting down. Because I've got nobody to interrupt [me].”

Finally, many participants stated that they were *mentally stimulated* by the sedentary behaviours they engaged in; these added to their motivation and enjoyment of those activities:

“Like knitting, sewing, you're counting a lot. Mentally when you're playing games, your stimulating your brains. So I mean, its keeping us mentally healthy longer…”

Extrinsic promoters to sedentary behaviour include *program access, awareness of programs, financial costs and culture/feeling accepted*. Decreased *program access* was identified. Study participants described how demand for fitness programs like Aqua-fit, often exceeded the space available. As a result, potential participants may feel frustrated and turn to sedentary activities at the center:

“Like here, we're at max…. sometimes we get people that, uh sign up for a course and they don't get in because you know, only twenty or thirty people are allowed in. There's only one program going because we don't have room where there's no, you know, um. No place to put people for another course. And, and so there are people that are turned away.”

There is also poor awareness of programs, including fitness classes that are available through the local senior's center. Participants describe the insufficient advertisements aimed at older adults living in the community: “You can advertise, but you don't see it, you don't hear. You need to hear it”. They also mentioned that certain places would be better for advertising.

“Well I think, I think that, that the people sure the doctors even places like the funeral places ‘cause that's where you go when you, when you have to bury somebody and you lose a partner. If they, if they advise people, ‘You know what? There's a lovely program in [town]. You know? That they offer different things when you're ready. When, when you feel you need to try them out…”

Finally, *financial* *cost* was identified as an extrinsic promoter of sedentary behaviour.

“I started out that way, but everybody doesn't do that. You know, a lot do in our little town, everybody had to volunteer, because there was not enough money to have programs, so the fire department had programs, the police department had them and we all volunteered… Money can be a barrier.”

Finally, the *role of cultural* and *feelings of acceptance* were identified as being extrinsic factors that impact the leisure domain. Some participants shared their beliefs that culturally based aging roles influence some older adults' decision to choose more typically sedentary activities:

“That's a difficult question because, a lot of cultures, don't encourage their older people to go out. And… especially if the older person has come… to join a young family… they feel that their responsibilities are not outside the house. Their interests are not outside the house. So it's very difficult …”

Related to the role of culture is the culture within the senior's centres that either makes new participants feel accepted or not:

“For me one of the…the big things was after spending a few years basically isolated, was the fear of coming, of not fitting in, of… you know, not belonging, um… It was very scary for me to come here…”

#### 3.2.2. Transportation Domain

Under the transportation domain, no intrinsic promoters were identified. Extrinsic promoters were *accessibility, transportation and weather*. With aging, participants identified decreased independence, particularly when accessing the community and this is related to laws and regulations of driving:

“Well I think that getting here sometimes people as they age, sometimes they, they can't drive anymore, their license at, you know, they have to take their, driver's license at eighty every two years.”

Due to these restrictions, there may be an increased reliance on public transit in order to participate in physical activities. This can be further influenced by the *accessibility* of public transit within the community.

“Well if you, if you have to take the bus, you get settled away. Because there is no bus routes.”

Compounded by issues of bad *weather,* older adults might find it even more challenging to engage in activities:

“When you got a bad weather day, you know, all of a sudden the attendance is down because if they have to wait for a bus. You know, an older person waiting for a bus is just not good. You don't wanna slip and fall and hurt yourself. It's that easy to get up again, you know. So I think getting here would be you know, transportation for, for older people is more difficult…”

These factors lie beyond the individual and influence decisions made about activity participation. If older adults are unable to access community based activities due to lack of transportation or inclement weather, they are forced to remain at home, and more likely to choose sedentary activities.

## 4. Discussion

The purpose of this study was to discuss the issue of sedentary time with older adults and gain insight into ways through which sedentary behaviour reduction strategies can be disseminated. The data used in this study are based on focus group interviews with community dwelling older adults at a number of seniors' centers located in a mid-sized Canadian city. Although the focus groups included discussions covering a variety of topics regarding thoughts and perceptions of sedentary behaviour [10], the current analysis also focused on the responses associated with the questions pertaining to programs and support needed to reduce sedentary time. The focus groups provided a rich dataset that helped us identify a range of intrinsic and extrinsic factors that either discouraged or promoted engagement in sedentary activities in each of the four domains of the ecological model. This is one of the first studies, to our knowledge, that examined factors that impact the choices of older adults to either participate in, or to avoid sedentary behaviours within the context of various sedentary domains.

There has been a call throughout the literature to continue to inform the development of public health messaging to reduce sedentary behaviours among older adults [15],[16],[17],[18],[19]. Despite these calls-to-action, little research has been done with older adults in an effort to obtain their perspectives for informing the knowledge translation process. Our data indicate that messaging may need to be developed for each domain and may need to consider intrinsic and extrinsic factors. On the basis of factors that promote and discourage sedentary behaviour as identified in this study, we have also highlighted implications for knowledge users and researchers to consider.

### 4.1. Intrinsic Factors Discouraging Sedentary Behaviour

Intrinsic factors discouraging sedentary behaviours are considered to be internal, or within the individual; these were identified in each of the four sedentary behaviour domains. For example, enjoyment was a factor that impacted decisions on engaging in sedentary activities in the areas of leisure, housework and occupation. Participants discussed the pleasure of engaging in activities such as fitness classes, gardening or volunteering at their community center or church. This is not surprising as previous studies have shown that enjoyment has a strong influence on attendance in both structured exercise classes as well as general activity groups [20]. Enjoyment also influences adherence to activities[21],[22] and has been noted to increase with higher levels of activity [23].

Another common intrinsic factor discouraging sedentary behaviour was motivation; this appeared in the leisure, transportation and occupation domains. It influenced other factors such as companionship, mental stimulation and physical health. Engaging in physical activities was associated with developing and maintaining friendships. This result is consistent with research looking at motivators regarding physical activity in older adult populations [24],[25]. Costello and colleagues (2011), conducted a qualitative study with older adults living in the community and determined that physically inactive individuals considered themselves physically active because their perceptions of physical activity were grounded in a social context [24]. Further, the level of engagement in physical activities may be predicted by social support [24]. This highlights the significant role that social relationships play, and their potential role as a cost-effective approach to increasing adoption and maintenance of physical activity [26]. While this social context promotes physical activity, it may also promote sedentary activities because they too have a strong social component.

Mental stimulation was identified as an intrinsic factor discouraging sedentary behaviour in the leisure domain, that is, older adults participated in physical activity to maintain cognitive function. The relationship between cognitive benefits and physical activity has been well-documented in the literature [27]–[30]. A longitudinal study of older adults found that physical activity was a means for developing friendships and that engaging in more cognitive activities, such as games and crossword puzzles, contributed to slowing cognitive decline in this population [30]. Kerr and colleagues reported a dose response relationship between the intensity of physical activity and cognitive functioning in older adults [29], such that participating in higher intensity activities resulted in better cognitive performance. Thus, while physical activity provides mental stimulation to older adults, care needs to be taken in promoting sedentary cognitive exercises, as older adults are clearly motivated to maintain cognitive function.

These findings point to the need to create sedentary time reduction strategies that are socially engaging to ensure enjoyment and thus maintenance of activities, and provide a cognitive stimulus to motivate older adults to adopt and maintain such activities. Current findings are supported by results reported in a study by Chang and colleagues, who found that empowerment interventions including knowledge acquisition, active participation, social support and exercise skills training were more effective than standard education in both decreasing sedentary behaviour and increasing physical activity [31]. Both studies highlight the importance of addressing multiple factors that discourage sedentary time and encourage physical activity.

### 4.2. Extrinsic Factors Discouraging Sedentary Behaviours

Extrinsic factors discouraging sedentary behaviours are elements that are beyond the control of the individual participating in the activity. This study found leisure and transportation domains were most impacted by extrinsic discouraging factors. For example, access to information on existing programs, as well as encouragement by physicians and peers to engage in such programs, directly impacted sedentary time.

Use of public transit also prevented accumulation of sedentary time, as it forced some activity by walking to bus stops and to seniors' centres. A recently published paper clearly demonstrated that the urban environment, specifically transportation, can significantly impact physical activity, and in turn reduce sedentary time[32]. These extrinsic discouraging factors have implications for neighbourhood development and the built environment. For example, building seniors centres and retirement homes on main bus routes may promote active transportation or use of public transportation, thereby reducing sedentary time.

### 4.3. Intrinsic Factors Promoting Sedentary Behavior

Intrinsic promoters of sedentary behaviour included factors such as physical health, aging attitudes, financial costs, lack of motivation, enjoyment, lack of companionship, and awareness of programs. In this study, the factors appeared to only impact the leisure domain and seemed to inform the decision making process ultimately resulting in engaging in sedentary behaviours. Similar findings were reported in a study that identified cost, weather and personal factors as barriers to physical activity [21]. These factors speak to the social and economic determinants of sedentary behaviours. Participants believed that aging attitudes might be an important factor as well. These attitudes are a deterrent to participation [33], and it has been suggested in the literature that those who have lower expectations for their aging self will also engage in lower levels of activity [34]. Older adults may also identify with positive role models, making peer-mentorship a potential option for sedentary time reduction [35].

It is clear that messaging around sedentary time reduction, access to programs, and psychosocial factors will be critical in impacting sedentary behaviours of older adults.

### 4.4. Extrinsic Factors Promoting Sedentary Behaviour

Extrinsic promoters of sedentary behaviours were factors impacting the leisure and transportation domains. Limited availability of physical activity programs was identified as a significant reason that many older adults did not join certain classes. This is in line with research on barriers to physical activity [21],[24],[36]. Many participants indicated that there was a lack of information from the greater community about available activities. Participants cited the importance of family doctors, funeral directors and the media in providing this information and promoting a physically active lifestyle [36]. Community based partnerships may provide additional opportunities for older adults to get involved. The Experience Corps program in Baltimore [37]–[39] placed volunteers in elementary schools for 15 hours per week to help students improve academic benchmarks. In comparison to a control group that was not volunteering, at the end of the 4-8 month period there were positive changes in physical activity levels, walking speed, and cognition. This is also in line with findings from the occupation domain around enjoyment and motivation for volunteering (intrinsic discouraging factors).

The role of culture and the feeling of acceptance were also identified as reasons that people chose to engage in more traditionally defined sedentary activities. These factors, external to individuals, tend to move older adults into less active, solitary activities. Thus, great consideration must be given to these extrinsic promoters as they may be simple to remove and may have a significant impact on older adults who are not currently socially engaged.

### 4.5. Physical Health in Discouraging and Promoting Sedentary Behaviour

Physical health was found to both discourage and promote sedentary time. As a discouraging factor, physical health served as motivation to engage in physical activities. For example, older adults with physical limitations were still participating in physical activity, such as walking, wherever possible.

As a promoter, physical health, specifically musculoskeletal issues pertaining to knees and low back, promoted engagement in sedentary activities, as physical activity was more challenging. This is in line with previous research that indicates that older adults with arthritis are more likely to be less active than those without arthritis [40],[41] despite there being significant benefit to engaging in regular physical activity [42]. Thus it is clear that sedentary reduction strategies must consider the range of physical abilities of older adults, and either cater to different functional levels, or be all encompassing in nature.

The results of this study can be used to inform further development of models such as the Ecological Model of Sedentary Behavior for older adults [8]. This model covers most factors relevant to most age groups, however, evidence from the current study, and future research, can be used to build upon this model. A finding from the current study that was particularly relevant to development of the model was in the occupation domain. Given that in 2011, seniors accounted for 14.8% of the population in Canada and is expected to rise[43], there is a need for a version of the Ecological Model that more accurately reflects this demographic. In addition, there are certain activities such as gardening that can be considered in both the leisure and housework domains, requiring further exploration in defining these areas.

There are some limitations to this study worth noting. The generalizability of this study is limited in that the participants are socially engaged older adults who were interviewed at local seniors' centers, of which they are members. Therefore, these results may not reflect the experiences of older adults if interviews were conducted in their homes or health care facility. Future research in this area should consider participation of individuals who are more isolated. Related to this, since our focus groups were conducted in seniors' centres, and since the original intent of the focus groups was not related to the Ecological Model for sedentary time in adults, data on the household domain are sparse. Future research is needed so that each domain can be specifically addressed. Another limitation of this study lies in the lack of sociocultural diversity in the participant population. The majority of participants were white women, calling for future research with a larger more sociocultural varied population to allow for comparisons between men and women and different sociocultural backgrounds. Finally, the interaction between sedentary time and physical activity was apparent through this analysis. Older adults assumed that reducing sedentary time meant increasing physical activity. This interaction should be further explored in future research to better understand how we can integrate messages pertaining to sedentary time and physical activity as they relate to health in older adults.

In conclusion, data from focus groups on older adults indicates that sedentary time reduction strategies and sedentary behaviour interventions could focus on the various domains outlined in the Ecological Model for sedentary time in adults. While our data were rich in some domains, future research is needed to better understand what messages are needed in the household domain. This study has identified a range of intrinsic and extrinsic factors that promote and discourage sedentary behaviour in older adults. Although some of these factors can be understood in the context of the Ecological Model for sedentary time in adults, as described by Owen (2011), there were some differences that can affect dissemination strategies. As a result, programs will have to consider intrinsic and extrinsic factors that promote and prevent sedentary behaviours. Programs will have to contain a social component, a mentally stimulating component, and will have to cater to older adults with different levels of physical functioning. Evidence-informed knowledge translation tools using quantitative and qualitative data need to be implemented and evaluated in future research.

## References

[1] Dogra S, Stathokostas L (2012). Sedentary behavior and physical activity are independent predictors of successful aging in middle-aged and older adults. J Aging Res.

[2] Pavey T, Peeters GMEE, Brown WJ (2012). Sitting-time and 9-year all-cause mortality in older women. J Sports Med.

[3] Gennuso KP, Gangnon RE, Matthews CE (2013). Sedentary behavior, physical activity, and markers of health in older adults. Med Sci Sports Exercise.

[4] Gianoudis J, Bailey CA, Daley RM (2014). Associations between sedentary behaviour and body composition, muscle function and sarcopenia in community-dwelling older adults. Osteoporosis Int.

[5] Manns P, Ezeugwu V, Armijo-Olivo S (2015). Accelerometer-derived pattern of sedentary and physical activity time in persons with mobility disability: Nation Health and Nutrition Examination Survey 2003-2006. J Am Geriatr Soc.

[6] Copeland JL, Clarke J, Dogra S (2015). Objectively measured and self-reported sedentary time in older Canadians. Prev Med Report.

[7] Golubic R, Martin KR, Ekelund U (2014). Levels of physical activity among a nationally representative sample of people in early old age: Results of objective and self-reported assessments. Int J Behav Nutr Phys Act.

[8] Owen N, Sugiyama T, Eakin EE (2011). Adults' sedentary behavior determinants and interventions. Am J Prev Med.

[9] Colley RC, Garriguet D, Janssen I (2011). Physical activity of Canadian adults: Accelerometer results from the 2007 to 2009 Canadian Measures Survey. Statistic Canada. Health Rep.

[10] Mcewan T, Tam-Seto L, Dogra S (2016). Perceptions of sedentary behavior among socially engaged older adults. Gerontol.

[11] Creswell J, Hanson W, Clark Plano V (2007). Qualitative Research Designs: Selection and Implementation. The Couns Psychol.

[12] Tremblay MS, Colley RC, Saunders TJ (2010). Physiological and health implications of a sedentary lifestyle. Appl Physiol Nutr Metab.

[13] Canadian Society for Exercise, Physiology (2013). Physical Activity and Health Training.

[14] Hsieh H-F, Shannon SE (2005). Three approaches to qualitative content analysis. Qual Health Res.

[15] Hsueh M-C, Liao Y, Chang S-H (2015). Are total and domain-specific sedentary time associated with overweight in older Taiwanese adults?. Int J Environ Res and Public Health.

[16] Judice PB, Silva AM, Santos DA (2015). Associations of breaks in sedentary time with abdominal obesity in Portuguese older adults. Age.

[17] Judice PB, Silva AM, Sardinha LB (2015). Sedentary bout durations are associated with abdominal obesity in older adults. J Nutr Health Aging.

[18] Burzynska AZ, Chaddock-Heyman L, Voss MW (2014). Physical activity and cardiorespiratory fitness are beneficial for white matter in low-fit older adults. PLoS ONE.

[19] Inoue S, Sugiyama T, Takamiya T (2012). Television viewing time is associated with overweight/obesity among older adults, independent of meeting physical activity and health guidelines. J Epidemiol.

[20] Hardy S, Grogan S (2009). Preventing disability through exercise: Investigating older adults' influences and motivations to engage in physical activity. J Health Psychol.

[21] Salmon J, Owen N, Crawford D (2003). Physical activity and sedentary behavior: A population-based study of barriers, enjoyment, and preference. Health Psychol.

[22] Stead M, Wimbush E, Eadie D (1997). A qualitative study of older people's perceptions of ageing and exercise: the implications for health promotion. Health Educ J.

[23] Dacey M, Baltzell A, Zaichkowsky L (2008). Older adults' intrinsic and extrinsic motivation toward physical activity. Am J Health Behav.

[24] Costello E, Kafchinski M, Vrazel JE (2011). Motivators, barriers, and beliefs regarding physical activity in an older adult population. J Geriatr Phys Ther.

[25] Park C-H, Elavsky S, Koo K-M (2014). Factors influencing physical activity in older adults. J Exerc Rehabil.

[26] Orsega-Smith EM, Payne LL, Mowen AJ (2007). The role of social support and self-efficacy in shaping the leisure time physical activity of older adults. J Leisure Res.

[27] Wischenka DM, Marquez C, Friberg Felsted K (2016). Benefits of physical activity on cognitive functioning in older adults. Annu Rev Gerontol Geriatr.

[28] Gill SJ, Friedenreich CM, Sajobi TT (2015). Association between lifetime physical activity and cognitive functioning in middle-aged and older community dwelling adults: Results from the Brain in Motion Study. J Int Neuropsychol Soc.

[29] Kerr J, Marshall SJ, Patterson RE (2013). Objectively measured physical activity is related to cognitive function in older adults. J Am Geriatr Soc.

[30] Robitaille A, Muniz G, Lindwall M (2014). Physical activity and cognitive functioning in the oldest old: Within- and between-person cogntive activity and pyschosocial mediators. Eur J Ageing.

[31] Chang AK, Fritschi C, Kim MJ (2013). Sedentary behavior, physical activity, and psychological health of Korean older adults with hypertension: Effect of an empowerment intervention. Res Gerontol Nurs.

[32] Sallis JF, Cerin E, Conway TL (2016). Physical activity in relation to urban environments in 14 cities worldwide: a cross-sectional study. Lancet.

[33] Ory M, Kinney Hoffman M, Hawkins M (2003). Challenging aging stereotypes. Am J Prev Med.

[34] Sarkisian CA, Prohaska TR, Wong MD (2005). The relationship between expectations for aging and physical activity among older adults. J Gen Intern Med.

[35] Horton S, Baker J, Cote J (2008). Understanding seniors' perceptions and stereotypes of aging. Educ Gerontol.

[36] Schutzer KA, Graves BS (2004). Barriers and motivations to exercise in older adults. Prev Med.

[37] Fried LP, Carlson MC, Freedman MM (2004). A social model for health promotion for an aging population: initial evidence on the Experience Corps model. J Urb Health.

[38] Tan EJ, Xue Q-L, Li T (2006). Volunteering: a physical activity intervention for older adults—the experience Corps® program in Baltimore. J Urb Health.

[39] Varma VR, Tan EJ, Gross AL (2016). Effect of Community Volunteering on Physical Activity: A Randomized Controlled Trial. Am J Prev Med.

[40] Bolen J, Hootman J, Helmick CG (2008). Arthritis as a potential barrier to physical activity among adults with diabetes--United States, 2005 and 2007. Morb Mortal Wkly Rep.

[41] Hootman JM, Murphy LB, Helmick CG (2011). Arthritis as a Potential Barrier to Physical Activity Among Adults with Obesity -- United States, 2007 and 2009. Morb and Mortal Wkly Rep.

[42] Fransen M, McConnell S, Hernandez-Molina G (2014). Exercise for osteoarthritis of the hip. Cochrane Database Syst Rev.

[43] Statistics Canada (2015). The Canadian Population in 2011: Age and Sex. Census Program.

